# Core Strength and Side-Hop Interlimb Asymmetries Are Associated with Subsequent Traumatic Knee Injury in Female Football Players

**DOI:** 10.3390/sports14090410

**Published:** 2026-09-17

**Authors:** Sofia Ryman Augustsson, Timmy Gustafsson

**Affiliations:** 1 Department of Sport Science, Linnaeus University, 39182 Kalmar, Sweden; 2 Unit of Physiotherapy, Department of Health, Medicine and Caring Sciences, Linköping University, 58185 Linköping, Sweden; timmy.gustafsson@liu.se

**Keywords:** injury discrimination, athletes, soccer, ACL injury, risk assessment, prospective cohort

## Abstract

The aim of this prospective cohort study was to examine the associations between muscle strength and functional interlimb asymmetries and the subsequent occurrence of traumatic knee injuries in female football players. A secondary aim was to evaluate the ability of asymmetry measures to discriminate between injured and non-injured athletes. A total of 104 female football players aged 16–26 years, representing elite-level clubs competing in the Swedish Elitettan and football academies, participated in this prospective cohort study. Baseline assessments included tests of muscle strength and functional performance, followed by a one-year prospective monitoring period with systematic registration of traumatic knee injuries (ACL, meniscal, collateral ligament, cartilage, and fracture injuries) and sport exposure. During follow-up, 21 players sustained a traumatic knee injury. Injured players demonstrated significantly greater interlimb asymmetry in quadriceps strength, core lateral flexor strength, side-hop performance, and hamstring-to-quadriceps (H:Q) ratio at baseline than non-injured players (all *p* ≤ 0.024). No significant differences were found for hamstring strength, hip external rotation strength, or single-leg hop for distance asymmetry. Core lateral flexor strength asymmetry (OR = 1.068, 95% CI: 1.013–1.126, *p* = 0.015) and side-hop asymmetry (OR = 1.061, 95% CI: 1.014–1.111, *p* = 0.011) remained statistically associated with subsequent traumatic knee injury in the exploratory multivariable model. Core lateral flexor strength asymmetry demonstrated acceptable discrimination (AUC = 0.756, 95% CI: 0.653–0.860), whereas side-hop asymmetry demonstrated modest discrimination (AUC = 0.652, 95% CI: 0.498–0.806). Exploratory sample-derived threshold values were 9.6% for core lateral flexor asymmetry and 29.9% for side-hop asymmetry. In conclusion, female football players who sustained a traumatic knee injury exhibited greater baseline interlimb asymmetries than uninjured players, particularly in quadriceps strength, core lateral flexor strength, side-hop performance, and H:Q ratio. However, only core lateral flexor strength asymmetry and side-hop asymmetry remained statistically associated in the exploratory multivariable model. These findings should be considered exploratory and require validation in larger independent cohorts.

## 1. Introduction

Female football involves repetitive high-intensity movements such as sprinting, cutting, deceleration, jumping, landing, and repeated single-limb loading, which place substantial demands on the knee joint [1,2]. Consequently, traumatic knee injuries are common and represent a major clinical concern in female football, with anterior cruciate ligament (ACL) injuries being among the most severe and extensively studied injury types [3,4]. Because traumatic knee injuries may lead to prolonged absence from sports, lengthy rehabilitation, and long-term functional consequences, identifying clinically feasible screening measures associated with injury occurrence remains important [5,6]. Although knee injuries in football are multifactorial, neuromuscular factors such as lower-limb strength, proximal control, and functional performance have received considerable attention as potentially modifiable contributors to injury risk [1,7]. In this context, interlimb asymmetries may be clinically relevant, as side-to-side differences in muscle strength or functional performance could reflect deficits in load tolerance, movement control, or sport-specific capacity [8]. Previous research has suggested that asymmetries may be associated with both impaired performance and increased injury risk, although findings remain inconsistent across populations, assessments, and injury definitions [9,10,11,12]. Consequently, the prospective relationship between interlimb asymmetry across multiple strength and functional performance measures and subsequent traumatic knee injury remains unclear.

Muscle strength around the knee, hip, and trunk may be particularly relevant for lower-limb control during football-specific movements. Reduced isometric hip abduction and external rotation strength has been shown to predict future non-contact ACL injury in competitive athletes [13]. Similarly, trunk or core strength may influence the ability to control whole-body position during dynamic tasks, while quadriceps and hamstring strength are important for knee joint loading and stability [7,14]. However, whether asymmetries in these strength measures are prospectively associated with subsequent traumatic knee injury in female football players remains unclear. Functional performance tests may provide additional information beyond isolated strength measures because they require coordinated force production, postural control, dynamic stability, and repeated single-limb loading [8]. Previous studies in female youth football players have highlighted the importance of integrating strength and functional performance assessments when evaluating characteristics related to athletic function and injury risk [15]. Tests such as the single-leg hop for distance and the side-hop test are commonly used to assess lower-limb function and side-to-side differences [16]. In particular, lateral hopping tasks challenge frontal-plane control, neuromuscular coordination, and dynamic knee stability, biomechanical factors that may be relevant to traumatic knee injury mechanisms [8,17]. However, the extent to which functional interlimb asymmetries can distinguish between female football players who do and do not sustain subsequent traumatic knee injuries has not been fully established.

While ACL injuries have received considerable attention because of their severity and long-term consequences, other traumatic knee injuries, including meniscal, collateral ligament, cartilage, and fracture injuries, are also common in football [18,19] and may result in substantial time loss from sports and impaired function. Consequently, the present study focused on traumatic knee injury as a composite outcome to capture the broader burden of serious knee injuries in female football players and to enable the prospective evaluation of potential injury-associated factors.

Therefore, the aim of this prospective cohort study was to examine the associations between muscle strength and functional interlimb asymmetries and the subsequent occurrence of traumatic knee injuries in female football players. A secondary aim was to evaluate the ability of asymmetry measures to discriminate between injured and non-injured athletes.

## 2. Materials and Methods

### 2.1. Study Design and Participants

A total of 104 female football players aged 16–26 years, representing elite-level clubs competing in the Swedish Elitettan and football academies, participated in this prospective cohort study (Table 1). Baseline assessments included tests of muscle strength and functional performance, followed by a one-year prospective monitoring period with systematic registration of injuries and sport exposure. Exclusion criteria comprised a previous traumatic knee injury and the presence of pain or discomfort during testing that could influence performance. All players were informed about the study’s purpose, procedures, potential benefits, and risks prior to participation. A partial cohort overlap existed with a previous prospective study from our research group examining associations between physical fitness test performance and traumatic knee injury [20]. Specifically, 25 of the athletes included in the previous cohort were also included in the present study. However, the present study addressed a distinct research question by examining interlimb asymmetries in muscle strength and functional performance, rather than absolute performance measures.

### 2.2. Procedure

Baseline assessments included muscle strength and functional performance tests, from which measures of interlimb asymmetry were derived. Prior to testing, players received information and instruction regarding the injury and exposure registration procedures. Data collection was performed by trained physiotherapists in a secluded room. Standardized verbal instructions and a visual demonstration were provided before each test. Demographic data, including age and football experience (years of participation), were collected using a questionnaire. Body height (cm) and body mass (kg) were measured using a stadiometer and a digital scale (Beurer SR BF2), respectively. Following the baseline assessment, injury occurrence and sport exposure (hours of training and match participation) were monitored prospectively over a one-year follow-up period.

### 2.3. Measurements

#### 2.3.1. Muscle Strength

Isometric muscle strength was assessed bilaterally using a handheld dynamometer (Commander Echo, JTECH Medical, Salt Lake City, UT, USA) secured with a fixation belt. Quadriceps, hamstring, core lateral flexor, and hip external rotation strength were evaluated according to previously described protocols [13,21]. Three maximal voluntary isometric contractions were performed for each muscle group, separated by 15 s of rest, and the highest value was used for analysis. Each maximal voluntary contraction was maintained for 5 s.

The highest value was selected to represent maximal performance capacity and to minimize the influence of unsuccessful attempts or occasional submaximal efforts, consistent with previously published testing protocols [21,22].

The test for quadriceps strength was performed in a seated position with the legs hanging from the edge of a treatment table and the knee positioned at approximately 90° of flexion [21]. A gait belt was attached to the leg of the treatment table and was used to stabilize the dynamometer during testing. The dynamometer was positioned just proximal to the ankle on the anterior surface of the leg. The player was instructed to extend the knee while the dynamometer was compressed against the belt. Players were instructed to stabilize themselves by holding the sides of the treatment table with their hands.

For the assessment of hamstring strength, the player was positioned in a prone position while one test leader stabilized the pelvis [21]. A belt was placed around the shank and secured to wall bars behind the player. The knee was maintained at 90° of flexion, and the dynamometer was positioned on the shank just proximal to the malleoli. The player was instructed to push the heel/shank against the belt as forcefully as possible by flexing the knee without lifting the pelvis from the table. Players were instructed to stabilize themselves by holding the ankles of the second test leader with their hands.

Core lateral strength was evaluated using the side-bridge test [21], with the dynamometer positioned at the iliac crest. The test was performed in a side-lying position with the knees extended and the upper foot placed in front of the lower foot. The player supported her body weight on the lower elbow and feet while lifting the hips to create a straight line through the trunk and lower extremities. The free arm was positioned with the hand placed on the waist. Both the left and right sides were assessed, and the player was given a three-minute rest period between tests.

Hip external rotation strength was measured using a testing position similar to the clam exercise described previously [13,23]. The player was positioned in side-lying on a treatment table, resting on the side opposite to the tested limb. The hips were flexed to 45° and the knees to 90°, with the tested limb positioned above the contralateral limb and the feet secured together with a strap. The player raised the knee of the tested limb away from the lower limb until the thigh was approximately parallel to the treatment table. A belt was positioned around the distal thighs without stretch or slack. The center of the dynamometer force pad was placed approximately 5 cm proximal to the lateral knee joint line. The player was instructed to push the thighs apart as forcefully as possible while maintaining contact between the heels.

These previously published protocols have demonstrated moderate-to-excellent reliability across the assessed muscle groups (knee flexors: ICC = 0.95, knee extensors: ICC = 0.80, hip external rotation: ICC = 0.99, core lateral flexors: ICC = 0.62) [13,21]. Strength values obtained from the right and left sides were subsequently used to quantify interlimb asymmetries. Hamstrings to quadriceps (H:Q) ratio was also calculated.

#### 2.3.2. Functional Performance

Functional performance asymmetries were evaluated using the 30-s side-hop test and the single-leg hop for distance (SLHD) test as described [16]. The side-hop test was performed separately on each limb, with players completing as many lateral hops as possible across a 40 cm distance in 30 s. The total number of repetitions was recorded for each limb. A side-hop repetition was considered valid when the player successfully cleared the 40-cm distance and landed with control on the tested limb.

During the SLHD test, players performed a maximal forward hop on a single limb, taking off and landing on the same limb while maintaining postural control for 2–3 s following landing. Arm swing was not permitted. Three trials were performed per limb, and the longest hop distance was retained for analysis. An SLHD trial was considered invalid if the participant failed to maintain the landing position for 2–3 s, touched the ground with the contralateral limb, or lost balance after landing.

The unilateral nature of both tests enabled the assessment of side-to-side differences in functional performance, and interlimb asymmetry was subsequently calculated from right and left limb performance outcomes. Both tests have previously demonstrated good test–retest reliability in athletes and are commonly used in knee injury assessment and rehabilitation (side-hop; ICC = 0.98, SLHD; ICC = 0.96) [16].

#### 2.3.3. Injury and Exposure Registration

Injury occurrence and exposure data, including training and match participation hours, were collected prospectively using a web-based questionnaire distributed via SurveyMonkey^®^ and adapted from a previously described registration form [20]. Players reported their training exposure, match participation, and any traumatic knee injuries on a biweekly basis throughout the follow-up period. Traumatic knee injuries sustained during football-related activities were recorded and verified by physical examination, MRI, and/or arthroscopy. A traumatic knee injury was defined as an injury to the ACL, other knee ligaments, menisci, articular cartilage, and/or fracture. Reminder emails were sent if no response was received within one week of each registration period.

### 2.4. Statistical Analysis

Statistical analysis was conducted using the IBM SPSS (IBM SPSS Statistics for Windows, Version 31.0. IBM, Armonk, NY, USA). Descriptive statistics are presented as mean ± standard deviation (SD). A sample size calculation was based on data from a previous study reporting a traumatic knee injury prevalence of approximately 30% [19]. The calculation indicated that approximately 80 athletes were required to detect a 10% between-group difference in asymmetry with 80% power. Therefore, at least 100 athletes were recruited to account for potential dropouts. The a priori sample-size calculation was designed for the planned between-group comparisons and was not intended to establish adequate sample size for the subsequent multivariable logistic regression model. Missing exposure data were handled using mean substitution [24]. A total of 43 of 2704 exposure reports were missing during the one-year follow-up, corresponding to a compliance rate of 98.4%, and were assumed to be missing at random [24].

Force values were normalized to body mass (N·kg^−1^) to allow comparison between players. Interlimb (side-to-side) asymmetry was calculated using the percentage difference between limbs, defined as ((higher value − lower value)/higher value) × 100, in accordance with previous recommendations [8]. Higher value represents the greater score obtained from either side. Independent-samples *t*-tests were used to compare interlimb asymmetry values between players who sustained a traumatic knee injury during the follow-up period and those who remained injury-free. Effect sizes were calculated using Cohen’s *d* and interpreted as trivial (<0.20), small (0.20–0.49), moderate (0.50–0.79), and large (≥0.80) [25]. Pearson product-moment correlation coefficients were calculated to examine the relationships among asymmetry variables and to assess potential multicollinearity. Correlation coefficients were interpreted as weak (*r* ≤ 0.39), moderate (*r* = 0.40–0.69), strong (*r* = 0.70–0.89) and very strong (*r* ≥ 0.90) [26]. Assumption checking was performed prior to analysis. Normality was assessed using the Shapiro–Wilk test and inspection of data distributions. Because some variables deviated from normality (*p* ≤ 0.02), supplementary non-parametric sensitivity analyses (Mann–Whitney U and Spearman rank correlations) were performed to evaluate the robustness of the findings.

To examine the association between asymmetry measures and the occurrence of traumatic knee injury during follow-up, multivariable binary logistic regression analysis was performed with injury status (injured/non-injured) as the dependent variable. The analyses were performed at the player level and players were classified as injured if they sustained at least one traumatic knee injury during follow-up. Additional injuries in already injured players did not contribute separately to the outcome. Given the limited number of injury events relative to the number of candidate predictors, a reduced exploratory multivariable model was specified. Only asymmetry measures demonstrating the strongest evidence of association in the primary analyses (Bonferroni-adjusted significance threshold: 0.05/7 = 0.007) were retained for inclusion in the model. Based on this criterion, quadriceps strength asymmetry, core lateral flexor strength asymmetry, and side-hop asymmetry were entered as independent variables. The logistic regression model estimated the association between baseline asymmetry measures and the probability of experiencing at least one traumatic knee injury during follow-up, rather than exposure-adjusted injury incidence or hazard. Results are presented as odds ratios (ORs) with 95% confidence intervals (CIs).

Receiver operating characteristic (ROC) curve analyses were conducted to evaluate the ability of asymmetry measures to discriminate between players who subsequently sustained a traumatic knee injury and those who remained injury-free. The ROC analyses were performed for variables demonstrating significant associations with injury status. Discriminatory ability was quantified using the area under the curve (AUC), with values interpreted as poor (0.50–0.69), acceptable (0.70–0.79), good (0.80–0.89), and excellent (≥0.90). Exploratory sample-derived thresholds were subsequently identified using the maximum Youden Index (J = sensitivity + specificity − 1). Sensitivity and specificity were calculated for these thresholds and are presented with 95% confidence intervals estimated using the Wilson score method for binomial proportions. Statistical significance was set at *p* < 0.05.

## 3. Results

### 3.1. Baseline Data and Injuries at Follow-Up

Baseline data are presented in Table 2. During the follow-up period, 21 players sustained a total of 26 traumatic knee injuries, comprising 11 ACL injuries, 7 meniscal injuries, 6 collateral ligament (MCL/LCL) injuries, and 2 cartilage injuries. Of the 26 injuries, 2 were classified as concomitant injuries. Seventeen injuries involved the right knee, whereas nine involved the left knee. The cumulative injury risk was 20.2%, and the injury incidence rate was 0.30 injuries per 1000 exposure hours based on 87,360 h of football exposure. Of the 26 traumatic knee injuries, 18 (69%) occurred during non-contact situations and 8 (31%) involved player contact.

### 3.2. Interlimb Asymmetries and Subsequent Traumatic Knee Injury

Significantly greater interlimb asymmetries were observed among injured players compared with non-injured players for quadriceps strength asymmetry (mean difference = 6.01%), core lateral flexor strength asymmetry (mean difference = 7.26%), side-hop performance asymmetry (mean difference = 11.33%), and H:Q ratio asymmetry (mean difference = 5.58%) (Table 3). No significant between-group differences were observed for hamstring strength asymmetry, hip external rotation strength asymmetry, or SLHD asymmetry (*p* ≥ 0.743). Supplementary non-parametric analyses yielded results comparable to those obtained from the parametric analyses and did not alter the interpretation of the findings. Effect size analysis indicated the largest between-group difference for side-hop asymmetry, followed by quadriceps strength asymmetry and core lateral flexor strength asymmetry. H:Q ratio asymmetry demonstrated a moderate effect size, whereas hamstring asymmetry, hip external rotation asymmetry, and SLHD asymmetry showed only small or negligible between-group differences (Table 3).

### 3.3. Independent Associations Between Interlimb Asymmetries and Traumatic Knee Injury

Predominantly weak-to-moderate relationships were observed between measures of interlimb asymmetry. The strongest association was found between hamstring strength asymmetry and H:Q ratio asymmetry (*r* = 0.596, *p* < 0.001). All other correlations were weak according to the predefined classification, although the association between quadriceps asymmetry and H:Q ratio asymmetry (*r* = 0.392, *p* < 0.001) was close to the threshold for a moderate correlation. In the exploratory multivariable logistic regression model, core lateral flexor strength asymmetry and side-hop asymmetry remained statistically associated with subsequent traumatic knee injury (Table 4). Specifically, each 1% increase in core lateral flexor strength asymmetry and side-hop asymmetry was associated with a 6.8% and 6.1% increase, respectively, in the odds of sustaining at least one traumatic knee injury during follow-up. In contrast, quadriceps asymmetry was not significantly associated with injury status.

### 3.4. Discriminative Ability of Interlimb Asymmetries

**Figure 1:**
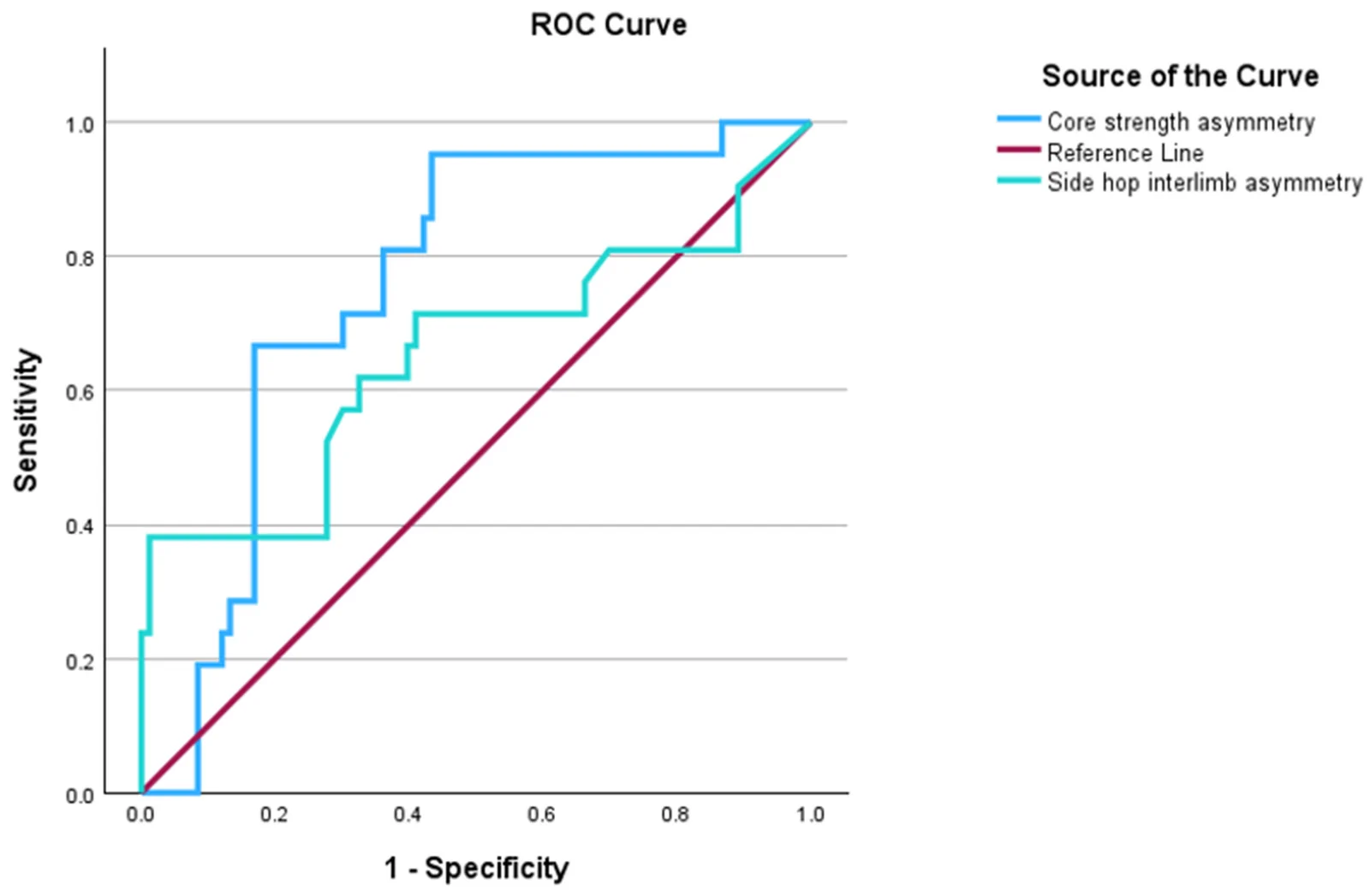
Receiver operating characteristic (ROC) curves for core lateral flexor strength asymmetry and side-hop asymmetry in relation to subsequent traumatic knee injury. The exploratory sample-derived threshold for core lateral flexor strength asymmetry was 9.6% (AUC = 0.756, 95% CI 0.653–0.860, sensitivity 95.2%, specificity 56.6%). The exploratory sample-derived threshold for side-hop asymmetry was 29.9% (AUC = 0.652, 95% CI 0.498–0.806, sensitivity 38.1%, specificity 98.8%).

ROC curve analysis revealed that core lateral flexor asymmetry showed acceptable discrimination between players who subsequently sustained a traumatic knee injury and those who remained injury-free (AUC = 0.756 (95% CI: 0.653 to 0.860)) (Figure 1). In contrast, side-hop asymmetry demonstrated modest discriminative ability (AUC = 0.652 (95% CI: 0.498 to 0.806)). The exploratory sample-derived threshold for core lateral flexor asymmetry was 9.6%, yielding a sensitivity of 95.2% (95% CI: 77.3% to 99.2%) and a specificity of 56.6% (95% CI: 45.9% to 66.8%). For side-hop asymmetry, the exploratory sample-derived threshold was 29.9%, corresponding to a sensitivity of 38.1% (95% CI: 20.8% to 59.1%) and a specificity of 98.8% (95% CI: 93.5% to 99.8%).

## 4. Discussion

The main findings of this prospective cohort study were that female football players who subsequently sustained a traumatic knee injury exhibited greater quadriceps, core, side-hop, and H:Q ratio asymmetries at baseline than players who remained injury-free. However, in the reduced exploratory multivariable model, only core lateral flexor strength asymmetry and side-hop asymmetry retained statistically significant associations with injury occurrence. Furthermore, core lateral flexor strength asymmetry demonstrated the strongest discriminatory ability, whereas side-hop asymmetry showed high specificity despite lower sensitivity. Together, these findings suggest that asymmetries in core lateral flexor strength and dynamic single-leg performance warrant further investigation in multifactorial models of traumatic knee injury.

One of the most notable findings was the association between core lateral flexor strength asymmetry and subsequent traumatic knee injury. Players who sustained an injury displayed substantially greater asymmetry in core lateral flexor strength than uninjured players, and core lateral flexor asymmetry remained statistically associated in the exploratory multivariable model. These findings are consistent with previous prospective studies showing that deficits in trunk neuromuscular control are associated with increased knee injury in athletes [7,14]. Adequate core function may contribute to the control of whole-body position during cutting, landing, and other high-demand football-specific actions. Conversely, impaired or asymmetrical core function may reflect neuromuscular characteristics that have previously been linked to proximal stability, lower-extremity alignment, and knee loading patterns [1]. However, the present study did not directly assess trunk kinematics, movement quality, or dynamic neuromuscular control. Therefore, any proposed biomechanical mechanisms should be regarded as hypotheses derived from previous research rather than explanations established by the current data.

The importance of trunk function for lower-extremity injury prevention has been highlighted previously [7,27,28]. Zazulak et al. [7] reported that deficits in trunk neuromuscular control predicted knee injury risk in female athletes, while intervention studies have shown that core training can improve trunk endurance and favorably alter lower-extremity biomechanics during side-step cutting, including reductions in knee valgus and adduction angles. Similarly, core strength training has been shown to improve trunk endurance and favorably alter lower-extremity biomechanics during side-step cutting, including reductions in knee valgus and hip adduction angles, both of which are linked to ACL injury risk [27]. Furthermore, video analyses of non-contact ACL injuries have demonstrated that excessive lateral trunk motion is associated with greater knee valgus during landing and cutting maneuvers, underscoring the importance of proximal control in maintaining lower-extremity alignment [29]. Collectively, these previous findings provide a plausible biomechanical rationale for why asymmetries in core muscle function may be associated with traumatic knee injury [28,29]. However, these mechanisms were not directly evaluated in the present study.

Side-hop asymmetry also remained statistically associated with injury occurrence in the exploratory multivariable model. This finding suggests that asymmetries in multidimensional functional performance may be associated with subsequent injury occurrence. The side-hop test requires repeated unilateral force production, dynamic postural control, deceleration capacity, frontal-plane stability, and rapid changes in direction, all of which are common features of situations in which traumatic knee injuries occur in football. Previous research has identified altered neuromuscular control and movement asymmetries as important contributors to lower-extremity injury risk in female athletes [1], and Myer et al. [30] suggested that asymmetrical movement strategies may increase joint loading and injury susceptibility. The present findings extend these observations by demonstrating that asymmetries in a simple field-based multidirectional hopping task were prospectively associated with traumatic knee injury in female football players. Approximately 70% of the injuries occurred during non-contact situations, indicating that non-contact injury mechanisms represented a substantial proportion of the observed outcomes. Nevertheless, because contact and non-contact injuries were combined in the primary analysis, the present findings cannot be interpreted as specific to non-contact injury mechanisms.

An additional finding was that side-hop asymmetry, but not SLHD asymmetry, was associated with injury occurrence. Although both tests are unilateral hopping assessments, they impose different neuromuscular demands. The SLHD primarily reflects maximal horizontal power production, whereas the side-hop test requires repeated force generation, deceleration, dynamic balance, and control in the frontal plane over multiple cycles of movement. Given that many traumatic knee injuries in football occur during cutting, landing, and multidirectional actions rather than during maximal power tasks, the side-hop test may better capture the neuromuscular control deficits and asymmetrical movement strategies that contribute to injury risk. This interpretation is consistent with previous suggestions that interlimb asymmetries are highly task-specific and that asymmetries observed in one test do not necessarily translate to another [8]. Consequently, multidirectional functional tasks may be more sensitive than linear performance tests for identifying deficits relevant to traumatic knee injury risk.

Interestingly, side-hop asymmetry demonstrated the largest between-group effect size (Cohen’s d = 0.995), yet only modest discriminative ability in the ROC analysis. This highlights the distinction between group-level associations and individual-level classification. A variable may demonstrate substantial differences between groups while still exhibiting considerable overlap between injured and non-injured athletes, limiting its usefulness for discriminating between injured and non-injured players at the individual level.

In contrast, core lateral flexor strength asymmetry demonstrated both an independent association with injury status and the strongest classification performance, suggesting that asymmetrical core muscle function may represent a particularly important characteristic among players who later sustain traumatic knee injuries.

The ROC analyses provided additional information regarding the ability of the identified asymmetry measures to distinguish between injured and non-injured players. A core lateral flexor asymmetry threshold of 9.6% yielded high sensitivity (95.2%), indicating that relatively few injured players in the present sample would have been missed using this threshold. However, the corresponding specificity was only 56.6%, indicating that a considerable proportion of uninjured players would also be classified as high risk when applying this threshold. In contrast, a side-hop asymmetry threshold of 29.9% demonstrated very high specificity (98.8%) but low sensitivity (38.1%), indicating that approximately six out of ten players who subsequently sustained a traumatic knee injury would not have been identified using this threshold alone. From a risk-identification perspective, high sensitivity may be advantageous because fewer at-risk athletes are missed, while high specificity may help identify athletes who are unlikely to sustain injury. The two measures may warrant investigation as candidate variables in future multifactorial injury-risk models [31]. Nevertheless, neither variable demonstrated sufficient accuracy to function as a stand-alone injury prediction tool.

Although quadriceps asymmetry and H:Q ratio asymmetry were significantly greater in injured players, these variables did not remain statistically significant after mutual adjustment. This may reflect shared variance between measures, limited statistical power, measurement error, or model instability. Recent findings in elite football populations further support the notion that the relevance of strength asymmetries depends on the specific outcome and assessment task rather than on asymmetry magnitude alone [32].

The moderate correlations observed between quadriceps asymmetry, side-hop asymmetry, and H:Q ratio asymmetry support the notion that these variables capture overlapping aspects of neuromuscular function. However, the relatively modest correlation coefficients indicate that each measure also provides distinct information regarding player characteristics.

Interestingly, no significant associations were observed for hamstring strength asymmetry or hip external rotation strength asymmetry. While some previous studies have reported associations between hip muscle deficits and knee injury risk [13,33], the present findings suggest that isolated hip external rotation strength asymmetry may be less relevant than asymmetries in core function or dynamic task performance. One possible explanation is that maximal hip strength tests do not adequately reflect the complex neuromuscular demands encountered during football-specific actions such as cutting, landing, and deceleration. Furthermore, the present study focused on strength asymmetry rather than movement quality, motor control, or hip kinematics during dynamic tasks, which may be more closely related to injury mechanisms. It is also possible that core strength asymmetry captured a larger proportion of the proximal neuromuscular deficits relevant to traumatic knee injury risk, thereby reducing the independent contribution of hip external rotation strength asymmetry in the multivariable model. These findings suggest that not all proximal asymmetries contribute equally to injury risk and that functional integration across the kinetic chain may be more important than isolated strength deficits.

Taken together, the findings support the growing body of literature suggesting that the relationship between interlimb asymmetry and injury risk is task-specific and highly dependent on the assessment method used [8,34,35]. Bishop et al. [8] highlighted that asymmetry magnitudes can vary considerably between tests and that asymmetries identified in one task are not necessarily reflected in another. Similarly, studies in football populations [34,36,37] have reported inconsistent associations between isolated strength asymmetries and injury occurrence, indicating that interlimb asymmetry should not be viewed as a universal injury-risk factor. Rather, its relationship with injury appears to be context-specific and influenced by the type of asymmetry assessed, the testing method employed, and interactions with other intrinsic and extrinsic risk factors. It should also be acknowledged that sports injuries are inherently multifactorial and cannot be explained by any single risk factor [38,39,40,41,42].

### 4.1. Strengths and Limitations

A major strength of this study is its prospective design, with baseline testing conducted before injury occurrence and a one-year follow-up period including systematic injury and exposure registration. This design reduces the risk of reverse causality and strengthens the interpretation of asymmetries as potential risk factors rather than consequences of injury. Furthermore, the inclusion of both strength-based and functional performance measures provided a comprehensive assessment of interlimb asymmetry.

Several limitations should be acknowledged. First, the number of injured players was relatively small (n = 21), which may have reduced statistical power and increased the risk of model instability. The relatively low number of injury events in relation to the number of predictors entered into the regression model may have increased the risk of model overfitting and unstable coefficient estimates. To mitigate this concern, the exploratory multivariable model was restricted to variables demonstrating the strongest evidence of association in the primary analyses, thereby reducing model complexity and improving the events-per-variable ratio.

Furthermore, the regression model and the ROC-derived thresholds were developed and evaluated within the same cohort and were not subjected to internal or external validation. Consequently, the reported associations and proposed threshold values should be regarded as exploratory and require confirmation in independent cohorts.

Second, interlimb asymmetry was assessed only at baseline, whereas asymmetry status may fluctuate throughout a competitive season due to training adaptations, fatigue, or minor injuries. In addition, although injury and exposure data were collected prospectively, the biweekly registration procedure relied on player self-report and may therefore have been subject to recall bias or incomplete reporting.

Third, although exposure was monitored prospectively, individual exposure time was not incorporated into the regression model. Since traumatic knee injuries are serious acute events that generally result in prolonged absence from sport, players were classified according to whether they sustained at least one injury during follow-up. Nevertheless, differences in exposure may have influenced injury opportunity and should be considered when interpreting the findings.

Fourth, different traumatic knee injuries were combined into a single outcome despite potentially having different underlying mechanisms and risk profiles. In addition, although most injuries occurred during non-contact situations, the outcome included both contact and non-contact injuries and no stratified analyses were performed. Fifth, multiple statistical comparisons were performed without formal adjustment for multiplicity, which may have increased the risk of Type I errors. However, the asymmetry variables were selected a priori based on the previous literature and predefined study objectives.

Furthermore, asymmetry values were derived from two limb-specific assessments and may therefore have been influenced by measurement error. Although the strength and functional performance tests used have demonstrated good reliability, it remains uncertain whether all observed asymmetry magnitudes exceeded measurement error. Consequently, asymmetry values, particularly those close to the proposed cut-off thresholds, should be interpreted cautiously. Additionally, the asymmetry calculation quantified only the magnitude of interlimb differences and not their direction. The relatively small number of injury events, particularly injuries affecting the left knee, limited the ability to explore potential relationships between asymmetry direction and the subsequently injured limb.

Finally, potential clustering effects at the club or academy level were not accounted for, and other potentially relevant intrinsic and extrinsic factors were not included in the analyses. Consequently, the observed associations should be interpreted within the context of the multifactorial nature of sports injuries.

### 4.2. Practical Implications

The present findings suggest that asymmetries in core lateral flexor strength and side-hop performance may represent potentially relevant markers associated with traumatic knee injury in female football players. Given the exploratory nature of the study and the absence of validation, these measures should not be considered ready for clinical screening. Rather, they may warrant inclusion in future multifactorial injury-risk models alongside other physical, biomechanical, and contextual factors.

## 5. Conclusions

Female football players who sustained a traumatic knee injury during the one-year follow-up demonstrated greater baseline asymmetries in quadriceps strength, core lateral flexor strength, side-hop performance, and H:Q ratio than players who remained injury-free. However, only core lateral flexor strength asymmetry and side-hop asymmetry remained statistically associated in the exploratory multivariable model. These findings suggest that asymmetries in core lateral flexor strength and side-hop performance may be associated with traumatic knee injury occurrence in female football players. However, the identified associations and proposed thresholds should be regarded as exploratory and require replication and validation in larger independent cohorts before clinical application can be considered.

## Figures and Tables

**Table 1:** Participant characteristics (n = 104).

Variables	Mean (SD)
Age (years)	17.5 (2.16)
Height (cm)	167.8 (5.3)
Weight (kg)	62.4 (6.8)
Experience (years)	10.3 (2.9)
Total training and	17.5 (5.3)
match load (hours/week)	
Competitive level	**n (%)**
Elitettan	28 (27)
Academy	76 (73)

**Table 2:** Descriptive characteristics of strength, functional performance, and relative strength measures in female football players (N = 104).

Variable	Mean (SD)
Quadriceps strength, R (N)	418.28 (84.14)
Quadriceps strength, L (N)	407.07 (82.13)
Hamstrings strength, R (N)	174.89 (31.91)
Hamstrings strength, L (N)	160.10 (31.41)
Hip external rotation strength, R (N)	219.64 (54.04)
Hip external rotation strength, L (N)	204.99 (57.89)
H:Q ratio, R	0.431 (0.104)
H:Q ratio, L	0.407 (0.112)
Core lateral flexor strength, R (N)	266.48 (65.83)
Core lateral flexor strength, L (N)	261.61 (61.42)
SLHD, R (cm)	132.61 (15.78)
SLHD, L (cm)	131.95 (16.10)
Side-hop test, R (repetitions)	41.79 (10.48)
Side-hop test, L (repetitions)	44.25 (9.96)
Relative quadriceps strength, R	6.77 (1.62)
Relative quadriceps strength, L	6.60 (1.63)
Relative hamstrings strength, R	2.83 (0.66)
Relative hamstrings strength, L	2.59 (0.60)
Relative hip external rotation strength, R	3.55 (0.94)
Relative hip external rotation strength, L	3.28 (0.85)
Relative core lateral flexor strength, R	4.30 (1.06)
Relative core lateral flexor strength, L	4.24 (1.10)

R = Right; L = Left; N = Newton; H:Q = hamstring-to-quadriceps force ratio; SLHD = Single-leg hop for distance; Relative strength values are normalized to body mass.

**Table 3:** Comparison of interlimb asymmetry measures between injured (n = 21) and non-injured (n = 83) female football players.

	Injured	Non-Injured		
Variable	Mean (SD)	Mean (SD)	*p*-Value	Cohen’s d
Quadriceps asymmetry (%)	14.10 (9.64)	8.09 (7.14) *	0.002	0.782
Hamstrings asymmetry (%)	12.36 (9.16)	11.11 (7.97)	0.534	0.152
H:Q ratio asymmetry (%)	18.31 (8.30)	12.73 (10.37) *	0.024	0.558
Hip external rotation asymmetry (%)	14.75 (12.76)	15.72 (11.81)	0.743	−0.08
Core lateral flexor asymmetry (%)	18.70 (6.97)	11.43 (10.22) *	0.003	0.751
Side-hop asymmetry (%)	22.54 (18.58)	11.21 (8.78) *	<0.001	0.995
SLHD asymmetry (%)	7.30 (4.59)	5.53 (5.17)	0.157	0.348

H:Q = hamstring-to-quadriceps force ratio. SLHD = Single-leg hop for distance. * = significant difference between groups.

**Table 4:** Multivariable logistic regression analysis of baseline interlimb asymmetry measures associated with subsequent traumatic knee injury in female football players.

Variable	β	SE	Wald	OR	95% CI	*p*-Value
Quadriceps asymmetry (%)	0.051	0.035	2.11	1.052	0.982–1.127	0.146
Core lateral flexor strength asymmetry (%)	0.066	0.027	5.862	1.068	1.013–1.126	0.015
Side-hop asymmetry (%)	0.059	0.023	6.408	1.061	1.014–1.111	0.011
Constant	−3.848	0.729	27.851	0.021		<0.001

Model statistics: n = 104; injured players = 21. Omnibus likelihood-ratio χ^2^ = 22.599, df = 3, *p* < 0.001. Cox & Snell R^2^ = 0.195; Nagelkerke R^2^ = 0.308. β = regression coefficient; SE = standard error; OR = odds ratio.

## Data Availability

The data that support the findings of this study are included in the article. The dataset supporting the findings of this study is not publicly available due to ethical restrictions but may be available from the corresponding author upon reasonable request and subject to ethical approval.

## Abbreviations

The following abbreviations are used in this manuscript:

ACL	Anterior Cruciate Ligament
MCL	Medial Collateral Ligament
LCL	Lateral Collateral Ligament
N	Newton
H:Q	Hamstring-to-Quadriceps
OR	Odds ratio
ROC	Receiver operating characteristic
AUC	Area Under the Curve
